# Lipedema Reframed: AFS Framework for Surgical and Transdisciplinary Management

**DOI:** 10.1007/s00266-026-05882-4

**Published:** 2026-05-11

**Authors:** Héctor Francisco Villa, Joaquim Muñoz, Alba Mayra Padilla-Correa

**Affiliations:** 1 Diagonal Clinic, Carrer de Sant Mateu, 24, 26, 08950 Esplugues de Llobregat, Barcelona Spain; 2 Jalisco Plastic and Reconstructive Surgery Institute “Dr. Jose Guerrerosantos”, Calzada del Federalismo Nte 2022, La Guadalupana, 44220 Guadalajara, Jalisco Mexico; 3 National Autonomous University of Mexico, Av. Universidad 3004, Copilco Universidad, Coyoacán, 04510 Ciudad de México, CDMX Mexico

**Keywords:** Lipedema, Adipoconnective Fragility Syndrome, Caveolae, CAVEOLIN-1, Lymphatic dysfunction

## Abstract

**Background:**

Lipedema has long been misclassified as a cosmetic concern or a subtype of obesity, leading to delayed diagnosis and suboptimal surgical outcomes. Growing molecular, histopathologic, and imaging evidence supports lipedema as a systemic disorder involving adipose tissue, connective matrix, vascular–lymphatic integrity, and neuroimmune regulation. To integrate these findings into a clinically actionable model, we introduce the concept of Adipoconnective Fragility Syndrome (AFS), framing lipedema as a multisystem condition with direct implications for surgical planning and perioperative management.

**Methods:**

A narrative review of the literature was conducted, integrating evidence from adipose biology, connective tissue pathology, endocrine signaling, vascular–lymphatic dysfunction, and pain neurobiology relevant to lipedema. Emphasis was placed on mechanisms with established clinical correlations, including disease progression, symptom severity, and surgical outcomes.

**Results:**

Lipedema tissue demonstrates early adipocyte hyperplasia, immune dysregulation, hypoxia-driven fibrosis, and abnormal sodium handling, resulting in a fragile adipoconnective microenvironment. Alterations in caveolar biology particularly involving CAVEOLIN-1 and its interaction with matrix remodeling pathways emerge as a key molecular mechanism contributing to hormonal hypersensitivity, vascular permeability, lymphatic overload, and pain. Within the AFS framework, these processes explain the resistance to weight-loss strategies, the propensity for recurrence, and the heterogeneity of surgical outcomes observed in clinical practice.

**Conclusions:**

Reframing lipedema as an Adipoconnective Fragility Syndrome provides a clinically relevant framework that enhances surgical decision-making rather than diminishing the role of surgery. This model supports earlier diagnosis, improved patient selection, individualized perioperative optimization, and structured long-term follow-up, aimed at reducing complications and recurrence. By linking molecular vulnerability to clinical behavior, AFS facilitates a more precise, multidisciplinary, and mechanism-based approach to the surgical management of lipedema.

**Level of Evidence V:**

This journal requires that authors assign a level of evidence to each article. For a full description of these Evidence-Based Medicine ratings, please refer to the Table of Contents or the online Instructions to Authors  www.springer.com/00266.

## Introduction

Lipedema is a chronic and progressive disorder characterized by disproportionate adipose tissue accumulation, pain, easy bruising, and resistance to conventional weight-loss strategies. Despite increasing recognition, it remains frequently underdiagnosed and misclassified as a cosmetic concern or a variant of obesity, leading to delayed treatment and inconsistent surgical outcomes [1–6]. These limitations suggest that lipedema cannot be fully explained as a localized fat disorder.

Surgical interventions, including liposuction and debulking procedures, can provide meaningful symptomatic and functional improvement. However, when lipedema is approached without consideration of its systemic biology, surgical benefits may be incomplete or short-lived, with persistent pain, edema, or disease progression [7, 8]. This highlights the need for a framework that links tissue-level pathology with clinical behavior and surgical response.

Emerging evidence indicates that lipedema involves coordinated alterations in adipose tissue, connective matrix, vascular–lymphatic integrity, endocrine signaling, and neuroimmune regulation [9–15]. These interactions help explain hallmark clinical features such as resistance to caloric restriction, diurnal swelling, venous insufficiency, and chronic pain [8, 12–14]. Lipedema is commonly classified according to anatomical distribution (types I–V) and clinical severity (stages I–IV), providing a practical framework for diagnosis, staging, and treatment planning **(**Table 1**)**.

**Table 1:** Integrated classification of lipedema by type and stage

Type	Affected areas	Stage I – initial	Stage II – intermediate	Stage III – advanced	Stage IV – complicated (Lipo-lymphedema)
Type I	Hips and buttocks	Mild volume increase in gluteal region and trochanters.	Subcutaneous nodules, padded skin in hips.	Hanging folds, severe gluteal contour asymmetry.	Persistent edema, lymphatic involvement of the inguinal region.
Type II	Buttocks to knees	Subtle disproportion in thighs, without significant pain.	Irregular skin with nodules; heaviness and frequent bruising.	Large fat masses in inner thighs and knees.	Distal lymphatic impairment, firm edema, positive Stemmer sign.
Type III	Buttocks to ankles	Symmetrical accumulation in calves, skin still normal.	Pain, bruising, early deformity; cuff sign evident.	Severe deformity, folds, gait limitation.	Secondary lymphedema, swollen feet, fibrosis.
Type IV	Arms (with or without lower limb involvement)	Fat accumulation in proximal arms; soft skin.	Painful nodules in the medial arm, easy bruising.	Adipose overhangs in triceps, mild-to-moderate functional limitation.	Arm lymphedema, fibrosis, chronic pain.
Type V	Legs (infrapatellar region only)	Rare; localized volume in lower legs.	Pain and bruising in calves without proximal involvement.	Severe dysmorphic changes in the sural region; walking difficulty.	Superimposed lymphedema, trophic skin changes in distal leg.

To integrate these observations into a clinically useful model, we introduce Adipoconnective Fragility Syndrome (AFS), framing lipedema as a systemic fragility disorder with direct implications for patient selection, surgical planning, perioperative optimization, and long-term management. Within this framework, alterations in caveolar biology, particularly involving CAVEOLIN-1, are proposed as a key molecular mechanism underlying hormonal hypersensitivity and vascular–lymphatic permeability [16, 17].

By reframing lipedema through the AFS construct, this review seeks to bridge molecular insight with surgical decision-making, providing a transdisciplinary roadmap to improve the safety, durability, and predictability of surgical outcomes.

### Clinical Translation of Adipoconnective Fragility Syndrome (AFS)

**Fig. 1:**
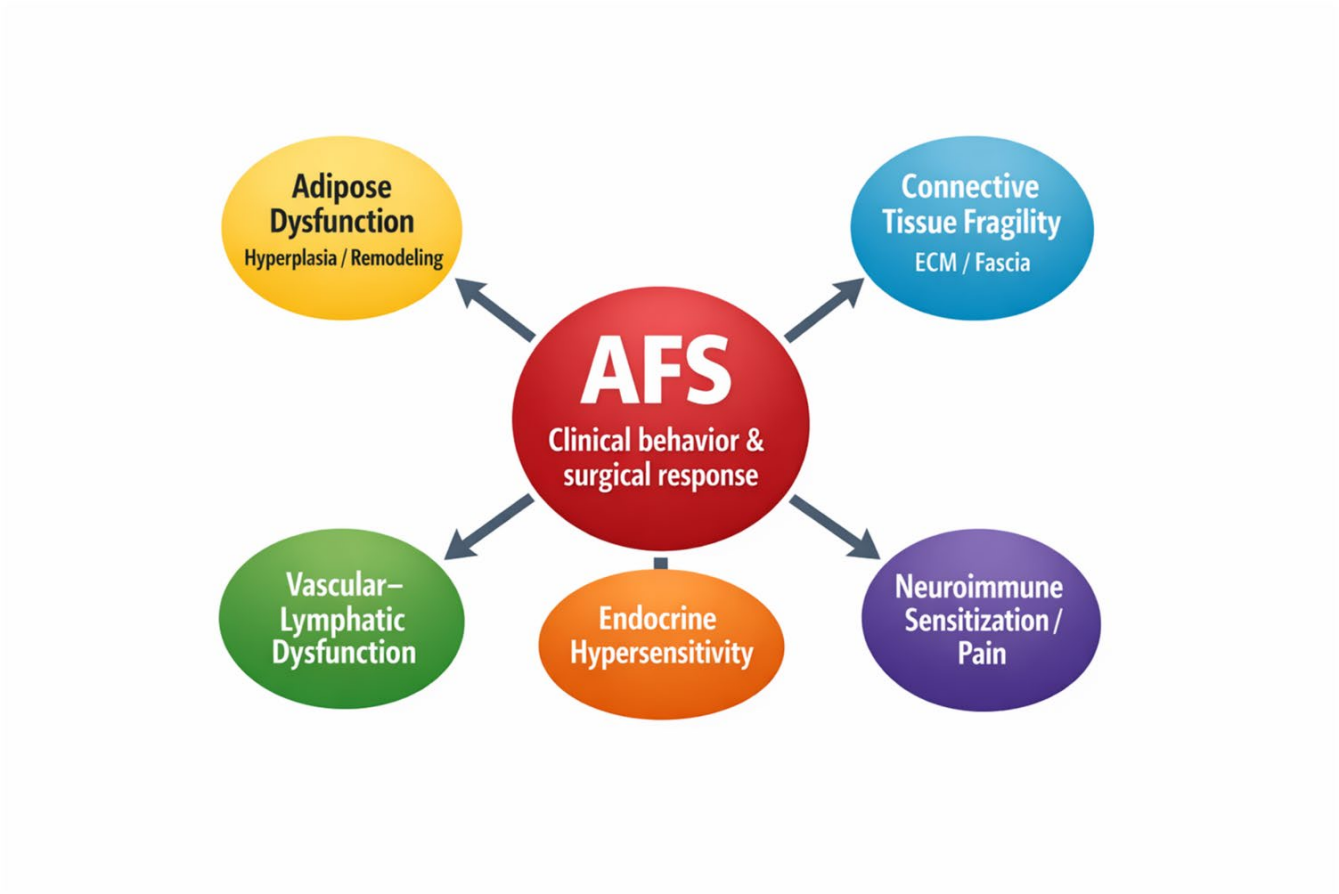
Adipoconnective Fragility Syndrome (AFS) as a clinically oriented framework for lipedema. AFS conceptualizes lipedema as a clinically relevant spectrum in which progressive involvement of adipose, connective, vascular–lymphatic, endocrine, and neuroimmune domains informs surgical risk stratification and perioperative decision-making

Reframing lipedema as an Adipoconnective Fragility Syndrome (AFS) provides a clinically actionable framework that translates molecular and tissue-level vulnerability into surgical decision-making. Rather than viewing lipedema as a localized fat disorder, AFS conceptualizes it as a systemic condition in which adipose dysfunction, connective tissue fragility, vascular–lymphatic compromise, and neuroimmune sensitization collectively influence surgical outcomes. This perspective has direct implications for patient selection, preoperative assessment, surgical technique, and postoperative management [7, 8]. Within this framework, lipedema can be understood as a spectrum of adipoconnective fragility in which progressive involvement of vascular–lymphatic, endocrine, and neuroimmune domains drives increasing surgical risk and variability of outcomes (Fig. 1).

#### ⁠ ⁠Patient Selection and Phenotyping

Within the AFS framework, not all patients with lipedema carry the same surgical risk. Clinical heterogeneity reflects the extent to which multiple adipoconnective domains are affected. Patients with predominantly adipose manifestations and preserved connective and lymphovascular integrity may respond predictably to surgical debulking. In contrast, those with widespread pain, venous insufficiency, orthostatic symptoms, hypermobility, or disproportionate bruising often exhibit advanced systemic fragility [12, 18–22].

Recognizing these phenotypes preoperatively allows surgeons to stratify risk, counsel patients realistically, and avoid attributing suboptimal outcomes to surgical technique alone. AFS-based phenotyping therefore shifts patient selection from volume-focused criteria toward biologically informed assessment [8, 17].

#### ⁠ ⁠Preoperative Assessment and Optimization

AFS supports a broader preoperative evaluation beyond standard anthropometric measures. In addition to clinical staging, assessment should include evaluation of venous and lymphatic function, pain phenotype, connective tissue laxity, and autonomic symptoms [8, 21, 23, 24].

Emerging diagnostic tools such as elastography, sodium-sensitive MRI, and biomarkers related to caveolar or extracellular matrix integrity may further refine risk stratification and longitudinal monitoring [13, 16, 25, 26]. While not yet standard of care, these modalities illustrate how AFS opens pathways toward mechanism-based diagnostics rather than purely morphological classification.

Optimization strategies may include hormonal modulation, compression therapy, lymphatic support, pain management, and fascial or musculoskeletal conditioning prior to surgery, particularly in patients with moderate to high AFS burden [7, 8, 20].

#### ⁠ ⁠Implications for Surgical Technique

Understanding lipedema as a fragile adipoconnective environment has implications for surgical planning and technique. In AFS, adipose tissue is embedded within a stiffened, fibrotic, and mechanically vulnerable matrix, increasing susceptibility to vascular injury, lymphatic disruption, and postoperative pain [9–11, 16, 27].

Gentle, staged approaches, meticulous tissue handling, and avoidance of excessive trauma are particularly important in patients with advanced systemic involvement. Rather than maximizing volume removal, surgical goals should emphasize functional improvement, symptom relief, and preservation of lymphovascular integrity [7].

AFS also explains why identical surgical techniques may yield divergent outcomes across patients: Success depends not only on procedural execution but on the underlying biological resilience of the adipoconnective system.

#### ⁠ ⁠Postoperative Management and Long-Term Follow-Up

AFS underscores that surgery represents one component of a longitudinal management strategy rather than a definitive cure. Persistent connective fragility, hormonal sensitivity, and neuroimmune activation may drive recurrence or symptom persistence if not addressed postoperatively [12, 20, 21, 28].

Structured follow-up incorporating compression therapy, lymphatic care, pain modulation, physical rehabilitation, and psychological support is therefore essential to sustain surgical benefits [22, 23]. Recognizing lipedema as a systemic fragility syndrome helps validate ongoing symptoms and prevents misinterpretation of postoperative pain or progression as surgical failure.

#### ⁠ ⁠AFS-Based Surgical Risk Stratification

To operationalize clinical translation, AFS allows classification into surgical risk phenotypes:

Low-risk AFS phenotype: Predominantly adipose involvement with minimal pain, preserved connective integrity, and limited vascular or lymphatic compromise. These patients tend to show predictable and durable surgical improvement.

Moderate-risk AFS phenotype: Multidomain involvement affecting two to three systems (e.g., adipose plus pain or venous dysfunction), requiring perioperative optimization and realistic expectation management.

High-risk AFS phenotype: Widespread systemic fragility with severe pain, connective tissue disorders, autonomic dysfunction, or lymphovascular insufficiency. In these patients, surgery should be staged, conservative, and integrated within multidisciplinary care [8, 12, 18, 20, 21]

**Fig. 2:**
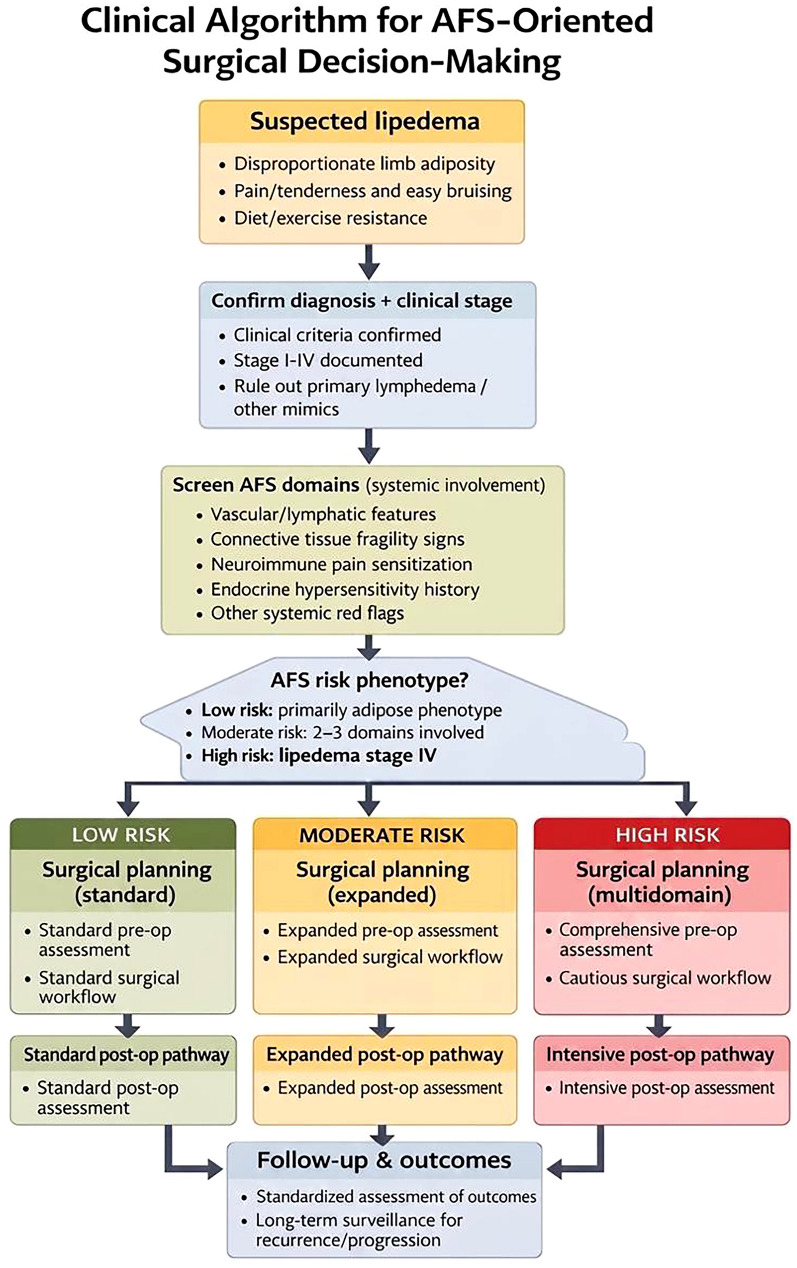
Clinical algorithm for AFS-oriented surgical decision-making in lipedema. Risk-stratified clinical algorithm linking AFS domain involvement to escalation of perioperative evaluation, surgical strategy, and postoperative follow-up

This stratification reframes surgical outcomes as biologically conditioned rather than technically determined, aligning expectations for both patients and surgeons. This risk-stratified approach is operationalized through a stepwise clinical algorithm that links AFS phenotyping to perioperative decision-making and escalation of surgical strategy **(**Fig. 2**)**.

### Clinical Relevance

By translating molecular vulnerability into clinical strategy, the AFS framework enhances surgical decision-making rather than diminishing the role of surgery. It provides a shared language for multidisciplinary collaboration, improves patient selection, and supports individualized perioperative care aimed at maximizing durability and safety of surgical intervention.

## Materials and Methods

This manuscript was developed as a narrative review based on a structured synthesis of the literature. Searches were conducted in PubMed, Scopus, and Web of Science for studies published between 2000 and 2025, using keywords related to lipedema, adipose and connective tissue biology, endocrine signaling, vascular–lymphatic dysfunction, caveolar mechanisms, and pain.

Original and translational studies, along with high-impact reviews, were selected for their relevance to the systemic and surgical aspects of lipedema. Articles focusing exclusively on obesity or lacking clinical or mechanistic relevance were excluded. No meta-analysis was performed. Selected principles of the PRISMA framework were applied to enhance transparency in literature identification and synthesis.

### Pathophysiology of Lipedema

Lipedema adipose tissue differs fundamentally from both healthy and obese fat. Rather than adipocyte hypertrophy, lipedema is characterized by early adipocyte hyperplasia, often initiated or accelerated by hormonal transitions, which explains its poor response to caloric restriction and bariatric interventions [9–11, 14].

At the tissue level, adipose expansion occurs within a fragile adipoconnective matrix marked by chronic low-grade inflammation, progressive fibrosis, and altered vascular–lymphatic integrity. These changes increase tissue stiffness and mechanical vulnerability, manifesting clinically as easy bruising, venous congestion, lymphatic overload, and pain [9–11, 16, 27].

Abnormal fluid handling represents a distinctive feature of lipedema. Increased intracellular sodium content within adipocytes promotes cellular swelling, hypoxia, and elevated interstitial pressure, contributing to diurnal edema and tenderness that are resistant to diuretic therapy [13, 26, 29].

Neuroimmune sensitization further amplifies symptom burden. Mechanical nerve compression within fibrotic tissue, together with altered ion-channel expression and neuroinflammatory signaling, underlies the high prevalence of chronic, often neuropathic pain in lipedema [12, 20, 21, 28].

At the molecular interface, dysregulation of caveolar biology, particularly involving CAVEOLIN-1, links hormonal hypersensitivity to membrane instability, vascular permeability, and lymphatic dysfunction. Within the AFS framework, this caveolar vulnerability provides a unifying mechanism connecting adipose expansion, fibrosis, fluid imbalance, and pain [16, 17, 30–32].

Together, these mechanisms define lipedema as a systemic adipoconnective disorder with direct implications for disease progression and surgical response.

## Discussion

Despite increasing recognition, lipedema continues to present substantial challenges in surgical practice due to delayed diagnosis, clinical heterogeneity, and variable postoperative outcomes. Traditional paradigms that conceptualize lipedema as a localized fat deposition disorder fail to explain the persistence of pain, edema, and disease progression observed even after technically adequate surgical intervention [1–8]. The Adipoconnective Fragility Syndrome (AFS) framework addresses these limitations by situating lipedema within a systemic biological context that directly conditions surgical response.

AFS integrates adipocyte hyperplasia, connective tissue fragility, vascular–lymphatic dysfunction, endocrine hypersensitivity, and neuroimmune sensitization into a unified model [9–16, 18, 19]. This perspective clarifies why caloric restriction and bariatric strategies are ineffective and why isolated debulking procedures may yield heterogeneous or transient results [5, 8–11]. By recognizing that surgical success depends not only on technical execution but also on the biological resilience of the adipoconnective system, AFS reframes outcome variability as biologically conditioned rather than surgically determined [8, 16, 17].

Importantly, the AFS framework does not diminish the role of surgery but refines its application. Liposuction and surgical debulking remain effective tools for symptom relief and functional improvement when integrated within a multidisciplinary strategy addressing lymphatic support, pain modulation, hormonal influences, and connective tissue vulnerability [7, 8, 20, 23, 24]. The clinical translation and risk stratification proposed in this review provide practical guidance for patient selection, expectation management, and perioperative planning, helping to reduce misinterpretation of persistent postoperative symptoms as technical failure [8, 12, 20, 21, 28].

By aligning surgical decision-making with underlying disease biology, AFS offers a pragmatic framework that improves the predictability and durability of surgical outcomes while validating the systemic nature of patient symptoms.

### Limitations and Future Directions

This manuscript is limited by its design as a narrative review and conceptual synthesis. While the Adipoconnective Fragility Syndrome framework integrates current molecular and clinical evidence, prospective and longitudinal studies are needed to validate risk stratification strategies and long-term surgical outcomes.

Emerging diagnostic tools targeting caveolar biology, extracellular matrix integrity, and tissue sodium content remain investigational and are not yet part of routine clinical practice. Future research should focus on translating these mechanisms into clinically actionable diagnostics and treatment algorithms.

## Conclusions

Reframing lipedema as an Adipoconnective Fragility Syndrome (AFS) provides a clinically meaningful paradigm that integrates adipose dysfunction, connective tissue fragility, vascular–lymphatic compromise, endocrine hypersensitivity, and neuroimmune sensitization into a unified framework. This approach moves beyond viewing lipedema as a localized fat disorder and explains the heterogeneity of symptoms, disease progression, and surgical outcomes encountered in clinical practice.

By linking underlying biology to surgical behavior, the AFS framework enhances patient selection, informs perioperative planning, and supports realistic expectation management. Rather than diminishing the role of surgery, this model refines its application, positioning surgical intervention as a key component within a broader, multidisciplinary strategy aimed at improving durability, safety, and patient-centered outcomes.

Recognizing lipedema as a systemic fragility syndrome represents a necessary shift toward earlier diagnosis, biologically informed treatment, and integrated long-term care. The AFS framework offers a shared clinical language that aligns patient experience with measurable biology and provides a foundation for future research and mechanism-based therapeutic strategies.
